# Development of a Multiplex Real-Time PCR Assay for the Newborn Screening of SCID, SMA, and XLA

**DOI:** 10.3390/ijns5040039

**Published:** 2019-11-02

**Authors:** Cristina Gutierrez-Mateo, Anne Timonen, Katja Vaahtera, Markku Jaakkola, David M Hougaard, Jonas Bybjerg-Grauholm, Marie Baekvad-Hansen, Dea Adamsen, Galina Filippov, Stephanie Dallaire, David Goldfarb, Daniel Schoener, Rongcong Wu

**Affiliations:** 1 PerkinElmer, 940 Winter St, Waltham, MA 02451, USA; galina.Filippov@perkinelmer.com (G.F.); stephanie.dallaire@perkinelmer.com (S.D.); david.goldfarb@perkinelmer.com (D.G.); Daniel.Schoener@perkinelmer.com (D.S.); rongcong.wu@perkinelmer.com (R.W.); 2 PerkinElmer, Wallac Oy, Mustionkatu 6, 20750 Turku, Finland; anne.timonen@perkinelmer.com (A.T.); katja.Vaahtera@perkinelmer.com (K.V.); Markku.Jaakkola@perkinelmer.com (M.J.); 3 Danish Center for Neonatal Screening, Statens Serum Institut, 2300 Copenhagen, Denmark; dh@ssi.dk (D.M.H.); jogr@ssi.dk (J.B.-G.); MABH@ssi.dk (M.B.-H.); DADN@ssi.dk (D.A.)

**Keywords:** Newborn Screening, SCID, SMA, XLA, DBS, real-time PCR, TREC, KREC, SMN1

## Abstract

Numerous studies have shown evidence supporting the benefits of universal newborn screening for primary immunodeficiencies (PID) and for Spinal Muscular Atrophy (SMA). We have developed a four-plex, real-time PCR assay to screen for Severe Combined Immune Deficiencies (SCID), X-linked agammaglobulinemia (XLA), and SMA in DNA extracted from a single 3.2 mm punch of a dried blood spot (DBS). A simple, high-throughput, semi-automated DNA extraction method was developed for a Janus liquid handler that can process 384 DBS punches in four 96-well plates in just over one hour with sample tracking capability. The PCR assay identifies the absence of exon 7 in the *SMN1* gene, while simultaneously evaluating the copy number of T-cell receptor excision circles (TREC) and Kappa-deleting recombination excision circles (KREC) molecules. Additionally, the amplification of a reference gene, *RPP30*, was included in the assay as a quality/quantity indicator of DNA isolated from the DBS. The assay performance was demonstrated on over 3000 DNA samples isolated from punches of putative normal newborn DBS. The reliability and analytical accuracy were further evaluated using DBS controls, and contrived and confirmed positive samples. The results from this study demonstrate the potential of future molecular DBS assays, and highlight how a multiplex assay could benefit newborn screening programs.

## 1. Introduction

Spinal Muscular Atrophy (SMA) is a group of hereditary diseases that progressively destroys motor neurons. It is one of the most common lethal recessive genetic disorders, and has an incidence of approximately 1/10,000 live births, and an estimated carrier frequency of approximately 1 in 57 [1]. It is characterized by significant motor disability, respiratory and nutritional compromise, and death in infancy or childhood in more than 50% of affected children. The majority of SMA cases are caused by defects in both copies of the survival motor neuron 1 gene (*SMN1*) on chromosome 5q. It is often classified into types 1 through 4, based on the age of onset, symptoms, and rate of progression. Children who display symptoms at birth or before six months typically have the lowest level of functioning SMN protein (type 1) and have significant motor neuron loss within the first six months of life. Types 2 and 3 typically have a later onset in childhood. Teens or adults generally have increasingly higher levels of SMN function, and they are classified as type 4. The neighboring *SMN2* genes can in part compensate for non-functional *SMN1* genes and hence high *SMN2* copy numbers often decrease the severity of the phenotype. 

Recently there have been significant advances in the therapeutic field, and two different drugs have been approved by the U.S. Food and Drug Administration (FDA) and became available to treat the condition. Nusinersen (Spinraza™), approved by the FDA in December 2016 and in Europe in June 2017, is an antisense oligonucleotide drug to treat individuals affected with SMA, including newborns [2]. More recently, in May 2019, Zolgensma^®^, an adeno-associated, virus vector-based gene therapy, received FDA approval as the first gene therapy for pediatric patients with SMA [3,4].

In the US, many of the tested conditions in newborn screening (NBS) are included in the Recommended Uniform Screening Panel (RUSP), which is a list of 35 core disorders and 26 secondary disorders that are recommended by the Secretary of the Department of Health and Human Services (HHS) for states to screen as part of their state universal newborn screening programs [5,6]. Disorders on the RUSP are chosen based on evidence that supports the potential net benefit of screening, the ability of screening tests for the disorder, and the availability of effective treatments. In 2010, Severe combined immunodeficiency (SCID) screening was added to the RUSP, allowing pre-symptomatic affected infants to be identified. More recently, evidence supporting the benefits of universal newborn screening for SMA was reviewed, and the condition was added to the RUSP in July 2018.

Since the inception of NBS more than fifty years ago, many laboratories face the challenge of screening for an expanding number of conditions [7]. The increase in the number of conditions included in newborn screening panels has an impact in reagent and consumable costs, labor, and sample availability. Public health newborn screening laboratories usually operate with a reduced budget and may have panels of more than thirty disorders to be tested on each sample, so it is essential that testing is done in a cost-efficient way and that it preserves as much real state from the dried blood spot (DBS) card as possible. 

Molecular testing in Newborn Screening is relatively new, with the first molecular test implemented in 2008 to screen SCID in newborns by quantitating T-cell receptor excision circles (TRECs) from dried blood spots using real-time PCR [8]. Real-time PCR is a technique widely used for routine SCID screening in NBS programs [9,10] and allows the ability to screen for multiple markers and/or conditions in a single analytical process. Another primary immunodeficiency disorder (PID), X-linked agammaglobulinemia (XLA), commonly caused by a mutation or deletion in the *BTK* gene which prevents the normal development of B lymphocytes and that results in a severe antibody deficiency, is being considered as a condition to be added into NBS panels in several countries [11,12,13]. Early diagnosis of PID patients facilitate early treatments, such as stem cell transplantation for SCID or immunoglobulin infusion therapy for XLA, which result in better outcomes [14,15].

With SCID and SMA being added in many NBS programs world-wide, and XLA being a strong candidate to be added into NBS panels, we developed a multiplex real-time PCR assay to allow the screening of infants with severe forms of PID manifested by T and B cell lymphopenia and identify the absence of exon 7 in the *SMN1* gene, which is present in approximately 96% of patients with SMA [16]. The assay amplifies four targets in a single PCR reaction; T-cell receptor excision circles (TREC), as a marker of SCID, Kappa-deleting recombination excision circles (KREC), as a marker of XLA, the exon 7 of the *SMN1/2* genes, and the RNase P (*RPP30*) gene, which is used as an internal control and to evaluate the quantity and quality of the DNA extracted from the 3.2 mm punches.

The 4-plex real-time PCR assay was used to evaluate population distribution in 3036 DNA samples isolated from de-identified leftover putative normal NBS specimens. Preliminary cutoffs were established based on this population. The assay performance and its potential clinical application was further evaluated by testing DBS reference samples with known copy numbers of the *SMN1* and *SMN2* genes, archived DBS specimens with confirmed diagnosis of SMA and contrived SCID and XLA positive DBS samples. The results from this study with a 4-plex real-time PCR assay demonstrate the potential of future molecular DBS assays.

## 2. Materials and Methods 

### 2.1. Dried Blood Spot Samples 

Leftover DBS specimens that have been obtained from the Danish Neonatal Screening Biobank (DNSB or DNS-Biobank) were used. These anonymized routine NBS DBS were collected between May 2013 and September 2013 (i.e., 6 years ago). Additionally, four archived newborn DBS specimens with confirmed diagnosis of SMA and age-matched putative normal specimens were also included in the sample set. This study was conducted in accordance with the Declaration of Helsinki and received ethic committee waiver for using anonymous DBS samples and associated data. The specimens were included in the study only if the parents/guardians of the newborn had not opted out the further use of the DBS used for NBS. The Danish ethics committee, De Videnskabsetiske Komiteer Region Hovedstaden, reviewed the study protocol and gave a waiver from ethics committee approval (protocol number 19000778, 27 February 2019). The Statens Serum Institut (SSI) gave a non-patient identifying specimen ID number all specimens.

A set of 28 reference DBS samples for SMA, with characterized *SMN1* and *SMN2* copy numbers, were provided by Biogen (Cambridge, MA, USA) and analyzed separately from the samples obtained from the Danish biobank.

### 2.2. Contrived and Control Dried Blood Spot Samples

Three DBS control samples were included in duplicates on each 96-well DNA extraction plate, and their DNA were extracted alongside the rest of the samples. The control samples were made spiking different concentrations of synthetic DNA targets into washed leukocyte depleted blood, which was spotted on 903 Protein Saver filter paper. The DBS controls mimic a normal newborn DBS (C3 controls), a newborn with close to cutoff levels of TREC and KREC (C2 controls), and finally a positive newborn (C1 control), with negligible levels of TREC, KREC, and SMN1. 

Other contrived samples were made for this study. Blood from an adult over 55 years of age was spotted on filter paper to mimic a SCID-like sample, as older individuals have very low or absent TREC counts. Contrived SMA, SCID, and XLA positive samples were made by spiking washed leukocyte depleted blood with SMA positive cells obtained from the NIGMS Human Genetic Cell Repository at the Coriell Institute for Medical Research (Repository ID GM23689). 

### 2.3. Punching and DNA Extraction

The punching was done into the wells of a 96-well PCR plate using a MultiPuncher™ instrument (PerkinElmer, Waltham, MA, USA). The 3.2 mm diameter punches underwent semi-automated DNA extraction using a JANUS^®^ G3 workstation instrument (PerkinElmer, Hopkinton, MA, USA) and an investigational use only prototype of the NeoMDx^TM^ DNA Extraction kit (PerkinElmer, Turku, FI). The semi-automated assay flow by the JANUS workstation included an initial wash of the DBS by adding 80 µL of the wash solution per well and incubating for 8 min at 25 °C, 700 rpm on a thermal shaker (INHECO^®^, Martinsried, Germany). After discarding the buffer, 80 µL of the elution solution was added to each well and incubated for 8 min at 25 °C, 700 rpm. Finally, after discarding the solution and waiting for the temperature of the thermal shaker to go to 70 °C, 80 more µL of the elution solution were added and the plate was incubated at 70 °C, 700 rpm for 30 min. The plates were then cooled down to 25 °C. 

### 2.4. Real-Time PCR

An investigational use only prototype of the NeoMDx PCR kit (PerkinElmer, Turku, Finland) was used, which consist in a PCR Reagent I (primer and probe mix) and a PCR Reagent II (PCR mix) that need to be combined in a 1:1 ratio. The PCR reagent I includes four primer pairs and five TaqMan™ hydrolysis probes, four of them labeled and one silent/unlabeled. The TREC, KREC, and RPP30 primers and probes have been described previously [17,18]. The SMN primers amplify the exon 7 of both the *SMN1* and *SMN2* genes and were described by Maranda et al. [19]. The labeled SMN1 probe was designed to target the SNP in exon 7 of the SMN loci, while the unlabeled SMN2 probe was designed to anneal to the homologous locus in the *SMN2* gene. Both probes incorporated several Locked Nucleic Acid (LNA™) nucleotide analogs (Exiqon, Woburn, MA, USA) that raised the melting temperature and increased the specificity of the probes to provide a superior discrimination between the two loci.

The master-mix preparation and the 384-well PCR set up was done semi-automatically by a JANUS G3 Mini Varispan™ Automated workstation (PerkinElmer, Hopkinton, US). Twelve µL of master-mix were dispensed into each well of the 384-well plate. The plate was then transferred into the JANUS^®^ G3 workstation instrument where 3 µL of extracted DNA per well from up to four 96-well extracted plates were consolidated semi-automatically into the 384-well plate. 

The PCR plate was sealed and run on a QuantStudio™ Dx real-time PCR instrument (Thermo Fisher, Waltham, MA, USA). The cycling conditions were 37 °C for 2 min, followed by 94 °C for 5 min, 40 cycles of 93 °C for 10 s, 60 °C for 30 s, and 69 °C for 40 s. The PCR run takes 1 h 28 min. Individual cycle thresholds for SMN1, TREC, KREC, and RPP30 were set and fixed for automated data collection.

### 2.5. Testing of Newborn DBS Samples

A population study and a preliminary cutoff determination was done analyzing 3036 anonymized leftover newborn DBS samples. The semi-automated workflow of the assay permits processing more than 1500 DBS samples from sample to result in less than 8 h with minimal hands-on time and sample tracking capability. The same PCR plate layout, including one set of controls (C1–C3) and one blank well (NTC or non-template control) encompassing the newborn specimens of each 96-well plate, was used throughout the study. 

### 2.6. Run and Sample Acceptance Criteria

The analysis started with a data validity check for each sample, including DBS controls in which the multicomponent data of the ROX reference dye and a baseline analysis is done. Any well/sample that fail to pass the data validity check was excluded from the subsequent data analysis. The second step was to do the validity check for DBS controls (included in duplicate on each 96-well DNA extraction plate). Any replicate of C1, C2, or C3 controls that have no RPP30 Ct value reported, which indicate a possible DBS loss or DNA extraction/PCR failure during the process, was excluded from the validity check. To accept a DNA extraction plate, the two replicates of the NTC controls and at least one replicate of each C1, C2, and C3 controls must have valid data and have met the pre-defined acceptance limits. Lastly, for the analysis of the de-identified newborn and contrived DBS samples, any samples that have RPP30 Ct > 28.4, which indicates that the DNA quantity or quality is not good enough to draw a conclusion due to the limitation of assay sensitivity and specificity, were reported as invalid or no-result for all three analytes.

### 2.7. TREC and KREC Copy Number Calculations and Statistical Analysis

A novel approach was used to semi-quantitate the TREC and KREC copy numbers. TREC and/or KREC concentrations in unit of copies/10^5^ cells were calculated based on the delta Ct (∆Ct) values between the two analytes and the *RPP30* reference gene. The following two formulas were used:

*TREC*: 2 × 2^−(*TREC* Ct−*RPP30* Ct)^ × 117,000 (1)

*TREC*: 2 × 2^−(*KREC* Ct−*RPP30* Ct)^ × 254,000 (2)

The first number 2 in the formula corresponds to the 2 copies of *RPP30* in a white blood cell, the second section of the formula determines the dilution factor for fraction of copies of TREC/KREC for each copy of *RPP30* in the cell; finally, the third part of the formula is an adjustment factor to correct for differences in *C*ts for equal TREC/KREC and *RPP30* copy numbers.

Statistical analysis was performed using R Studio (Version 1.2.1335). Descriptive statistics (e.g., *n*, mean/median, SD, and range) were calculated for continuous variables. From normal newborn population distribution data (on copies/10^5^ cells scale) lower percentiles (e.g., 0.1%, 0.5%, and 1%) were calculated for TREC and KREC, and were then used as cutoff(s). For SMA, cutoff data was divided into screen positive and screen negative results based on predefined cutoff (i.e., the pre-defined Ct value 31.2). The specimens with confirmed diagnosis of SMA were used to confirm that the predefined SMA cutoff was set at correct level.

## 3. Results

### 3.1. Presumed normal Newborn Samples

Archived anonymized presumed normal specimens were used for determination of the newborn population distribution and to establish the cutoff for each of the targets. A cohort of 3036 newborn DBS was tested. A total of 35 96-well plates were extracted and consolidated in 10 384-well plates for real-time PCR runs. All the plates passed the data validity check criteria, so there were no excluded plates/runs. A set of three DBS controls (C1–C3) and one NTC or blank well was included on each 96-well plate in duplicates so data for 70 replicates per control was obtained. There were 13 samples displaying either no Ct values for RPP30 or Ct > 28.4, indicating either DBS loss or sub-optimal DNA extraction/amplification. These samples were excluded from the analysis. The total sample exclusion rate was 0.4% (13/3036).

**Figure 1:**
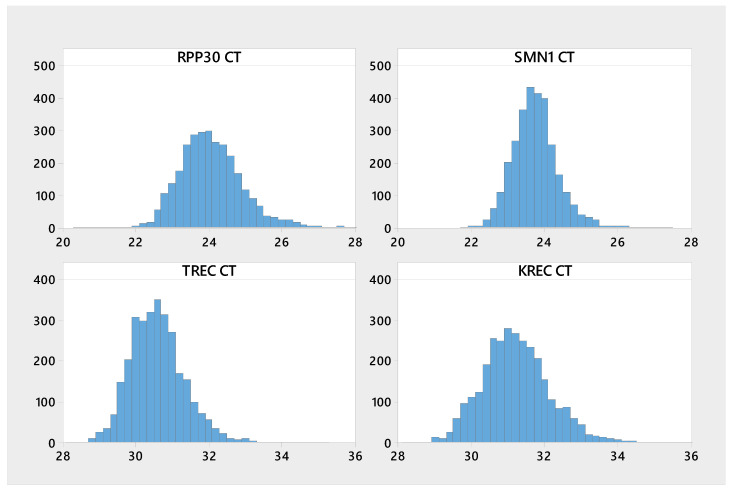
Histogram of RPP30, SMN1, TREC, and KREC Cts.

**Figure 2:**
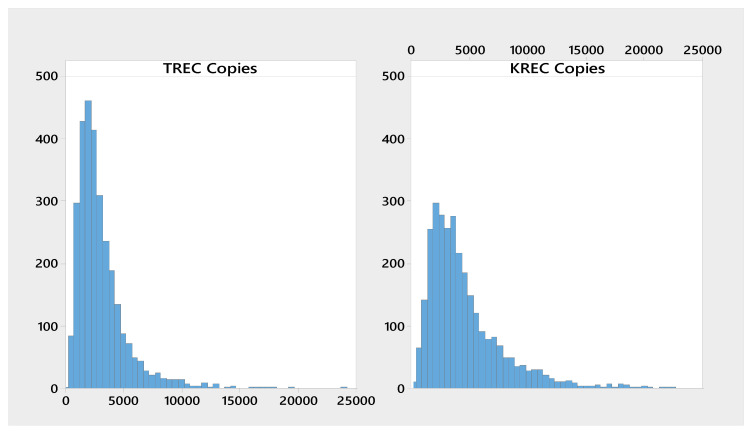
Histogram of TREC and KREC copy numbers/10^5^ cells.

Relevant descriptive statistics including sample size, mean, standard deviation, coefficient of variation, and range, were calculated for Ct values as well as TREC and KREC copies/10^5^ cells and Ln copies. The results are presented in Table 1. Figure 1 represent the histograms for the Cts of all four analytes, while Figure 2 represents the copies/10^5^ cells for TREC and KREC. The mean Ct for TREC was 30.6, while the median was 30.5. For KREC the Ct mean and median was 31.2, approximately 0.7 cycle higher than for TREC. Low percentiles (0.1%, 0.5%, 1%) were calculated for TREC and KREC copy numbers/10^5^ cells and shown in Table 2. The cutoff values corresponding to the 0.5 percentile was used to classify the samples as screening positives and negatives. These preliminary cutoffs produced an initial positive result in 30 samples, 15 screen positives for TREC, and 15 screen positives for KREC, which would have required repeat testing in routine newborn screening.

High percentiles (99%, 99.5% and 99.9%) were calculated for SMN1 Cts and displayed in Table 3. All the putative normal SMA samples had Cts ≤ 27.4. A SMN1 cutoff at Ct 31.2 was proposed for qualitative interpretation of SMA.

### 3.2. Reference, Contrived, and Confirmed Positive Samples

The analytical specificity was evaluated testing a set of 28 reference DBS samples for SMA, with known *SMN1* and *SMN2* copy numbers determined by digital PCR. This set included five SMA carriers and 23 SMA positive samples homozygous for the exon 7 deletion and *SMN2* copy numbers ranging from one to four copies. Additionally, four newborn DBS samples with confirmed diagnosis of SMA, 34 contrived SCID-like with low or absent TREC, and four contrived SMA, SCID, and XLA positive samples were included in the analysis. All the samples were tested against the pre-determined cutoffs of 231 copies/10^5^ cells for KREC and 380 copies/10^5^ cells for TREC, and the qualitative cutoff at Ct 31.2 for SMN1.

**Figure 3:**
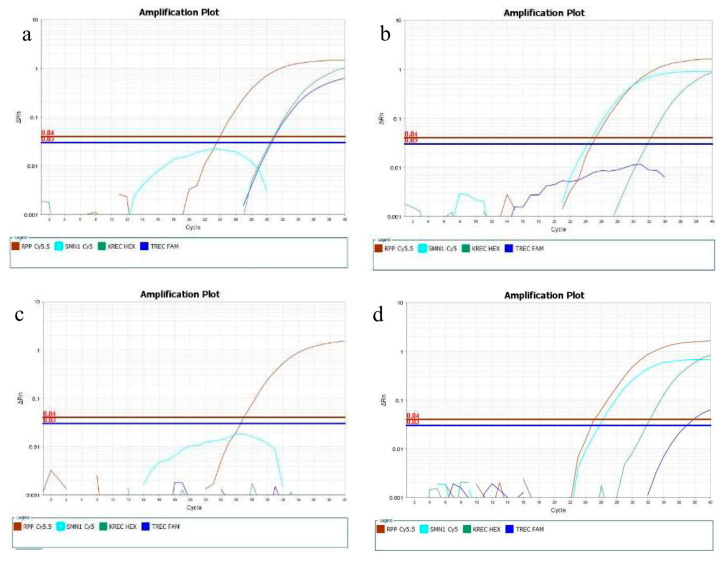
Amplification plots for: (**a**) A reference SMA positive sample with three copies of the *SMN2* gene showing no amplification of the SMN1 locus; (**b**) an individual over 55 years old (SCID-like) showing no amplification for the TREC locus and normal amplification for the other three targets; (**c**) a contrived SMA, SCID, and XLA positive sample showing amplification only for RPP30. (**d**) A reference SMA carrier sample showing robust amplification of the SMN1 locus.

Twenty-six out of the twenty-seven SMA positive samples were detected (23 SMA reference samples and 3 confirmed positive newborn samples), with a Ct above 40 in all of them. One confirmed positive newborn sample displayed normal amplification for the *SMN1* gene, with a Ct of 25.32. This newborn was a compound heterozygous for the exon 7 deletion and the c.422_428del mutation. The five SMA-carrier samples had SMN1 *C*t ranging from 24.8 to 26.6, and thus they were reported as presumptive normal, as expected. All the SCID-like samples displayed no TREC amplification or copies/10^5^ cells above the cutoff. The results for the contrived SMA, SCID, and XLA positive samples were also as expected, as no amplification was observed for the TREC, KREC, and SMN1 loci in any of the samples analyzed. Examples of the amplification plots from reference and contrived samples are shown in Figure 3.

## 4. Discussion

The goal of newborn screening is to detect potentially fatal or disabling conditions in newborns as early as possible so they can be treated successfully, reducing lifelong damage and mortality. Our ability to detect and treat more conditions is expanding due to a better understanding of the genetic basis of disease and the advances in technology and therapeutics. This results in more conditions being added to NBS panels. We have developed a multiplex assay to streamline the testing of three conditions which are already or will likely be included in NBS screening panels across the world [20,21,22]. This semi-automated assay can process a variable number of DBS samples, ranging from 32 to more than 1500, in less than eight hours and with minimal hands-on time. This flexibility permits the assay to be used in low and high throughput NBS laboratories, which may need to process more than a thousand of samples a day [10].

The 4-plex real-time assay includes primers that amplify the exon 7 of both the *SMN1* and *SMN2* genes [19], which are highly homologous, with only five nucleotides different between them [23]. SMA patients have at least one copy but possibly multiple copies of the *SMN2* gene, so the main challenge for the screening of the *SMN1* exon 7 deletion is to avoid false negatives due to the cross-reaction with the *SMN2* gene. We used two different strategies to overcome this challenge: (1) The SMN1 probe was designed to include LNA™ nucleotides that increased the specificity of the probe. This strategy has been previously described for a probe located in the intron 7 of the *SMN1* gene [17], but the intron 7 target may result in false positives due to gene conversions caused by intragenic recombination between the *SMN1* and *SMN2* genes [24,25]; (2) we included an unlabeled SMN2 probe that had a single nucleotide difference with the SMN1 probe and which also included LNA™ nucleotides. The unlabeled SMN2 probe anneals to the SMN2 locus with higher efficiency than the SMN1 probe, so it prevents the SMN1 probe to bind to the homologous SMN2 locus even in the absence of its higher homologous target, the exon 7 of the *SMN1* gene (i.e., in samples homozygous for the exon 7 deletion). Similarly, the labeled SMN1 probe will bind to the SMN1 locus more efficiently than the unlabeled SMN2 probe, so the possibility of false positives due to the silent SMN2 probe hampering the annealing of the SMN1 probe to its target sequence is extremely low. In fact, no SMA positives were detected in this cohort of over 3000 presumptive normal newborns. These two approaches provided a superior discrimination between the two loci. In fact, none of the 26 SMA positive samples with the exon 7 deletion displayed any amplification of the *SMN1* gene (i.e., Ct > 40 cycles) so no non-specific signal was produced by the *SMN2* gene, even when the assay was challenged by samples with four or more *SMN2* copies, in seven out of the 23 SMA positive reference samples. Although the absence of both copies of the *SMN1* gene is reported to be a very reliable assay for the molecular diagnosis of SMA, approximately 4% of SMA patients have other types of mutations that will not be detected by homozygous deletion testing [16]. Therefore, the clinical sensitivity is approximately 96%. However, SMA screening methods have high (100%) positive predictive value, and no false positives have been reported when screening for homozygous deletions of exon 7 [26,27].

We propose a qualitative test for SMA. The cohort of 3036 putative normal SMA samples naturally included ~53 carriers based on the reported carrier frequency in the population [2]. The SMN1 Ct values obtained with this assay ranged between 21.8 and 27.4. These results correspond well with the data derived from the five reference SMA carrier samples, which had SMN1 Cts ranging from 24.8 to 26.6. Considering that there is an overlap between the SMN1 Ct values in normal and SMA carrier samples (data not shown), it is very challenging to identify carrier status based on real-time PCR and specifically on SMN1 Ct values only. This works in the benefit of NBS programs that do not allow carriers to be identified or reported [27]. In consideration of a balance between sensitivity and specificity based on the population data, we set a SMN1 cutoff at Ct 31.2 for the qualitative interpretation of SMA. All the 26 SMA positives samples with the homozygous deletion of exon 7 were correctly identified using this cutoff.

In this study we report preliminary cutoffs for TREC and KREC at 380 and 231 copies/10^5^ cells based on the 0.5% percentiles in a Danish population of 3023 newborn specimens. The distributions of TREC and KREC Ct values followed normal distributions, with medians at 30.5 and 31.2 *C*ts, respectively. Simultaneous screening of SCID and XLA has been proposed as a more comprehensive approach to screen newborns for PID [28], as it allows the identification of clinically relevant types of PID that would require immediate attention, but could be missed if we only tested TREC molecules [13,29]. These conditions include severe forms of B cell deficiencies such as XLA, with an approximate incidence of 1:100,000, but also some patients with late-onset adenosine deaminase deficiency (ADA) and purine nucleoside phosphorylase (PNP) deficiencies [29].

The preliminary cutoffs were tested for clinical relevance against three newborns confirmed positive SMA samples homozygous for the exon 7 deletion, 23 reference SMA positive samples, 5 reference SMA carriers, 34 contrived SCID-like samples, and 4 contrived SMA, SCID, and XLA positive samples. All these samples except for the SMA carriers were correctly identified as screen positives based on the preliminary cutoffs. In the sample set of 3036 putative normal newborns and according to the established cutoffs, any sample with RPP30 Ct > 28.4 (13 samples in our cohort), SMN1 Ct > 31.2 (0 samples), TREC copy numbers/10^5^ cells below 380 (15 samples), or KREC below 231 copy numbers/10^5^ cells (15 samples) would have been repeated in a newborn screening setting. This represents a 1.4% assay repeat rate (43/3036), which corresponds well with existing literature [8,30]. Actual newborn SCID and XLA confirmed positive samples would be necessary to validate the respective TREC and KREC cutoffs in this population.

The use of DBS control samples in each processed plate is important, as it serves as a process control and allows monitoring the DNA extraction and amplification performance of the real-time PCR assay. Similarly, the inclusion of the reference gene *RPP30* in the multiplex, which is well-established in many newborn screening assays [30], is very useful not only to evaluate the quantity and quality of the DNA from the 3.2 mm punches, but also as an internal control to distinguish positive samples (with low or absent levels for one of the three targets) from samples with extraction/amplification issues or failures. Additionally, the ∆Ct values between the TREC/KREC analytes and the *RPP30* gene is a novel strategy to semi-quantitate the TREC and KREC molecules without the need of external calibrators, which consume reagents and plate real-state. Additionally, calibrators are typically made from serial dilutions of plasmids or other synthetic DNA molecules, which may be shifting over time [8,31,32].

We have reported population distribution data and preliminary cutoff determination for the TREC, KREC, and SMN1 targets using this novel 4-plex assay. Further research is needed to validate the assay and prove its usability in discriminating between normal and affected SMA, SCID, and XLA samples, as well as its ease of integration in a newborn screening laboratory setting. This study demonstrates the potential of a multi-targeted, molecular DBS real-time PCR assay, and provides a cost-effective and semi-automated solution to test for SCID, SMA, and XLA in low to high throughput newborn screening programs. The value of multiplexing these three conditions is significant as it reduces labor, costs, and sample use, all of them key factors in the ability to test for an expanding number of disorders in newborn screening.

## Figures and Tables

**Table 1:** Descriptive statistics for the Ct values of all four analytes.

Analyte	*n*	Mean	Median	SD	CV	Min	Max
KREC	3020	31.2	31.2	0.97	3.11	28.3	39.3
RPP30	3023	24.1	24	0.88	3.66	20.4	28.4
SMN1	3023	23.7	23.7	0.63	2.64	21.8	27.4
TREC	3022	30.6	30.5	0.76	2.48	28.2	35.1

**Table 2:** Low percentiles for T-cell receptor excision circles (TREC) and Kappa-deleting recombination excision circles (KREC) copies/10^5^ cells.

Analyte	*n*	Mean	Median	P0.1	P0.5	P1
∆Ct KREC-RPP30	3023	4790	3590	34.8	231	466
∆Ct TREC-RPP30	3023	3220	2500	218	380	518

**Table 3:** SMN1 Ct percentiles.

Analyte	*n*	CT Mean	Median	P 99.9	P 99.5	P 99.0
SMN1	3023	23.7	23.7	27.1	25.9	25.5
